# Effect of Tricalcium Magnesium Silicate Coating on the Electrochemical and Biological Behavior of Ti-6Al-4V Alloys

**DOI:** 10.1371/journal.pone.0138454

**Published:** 2015-09-18

**Authors:** Hossein Maleki-Ghaleh, Masoud Hafezi, Mohammadreza Hadipour, Ali Nadernezhad, Ermia Aghaie, Yashar Behnamian, Noor Azuan Abu Osman

**Affiliations:** 1 Faculty of Materials Engineering, Sahand University of Technology, Tabriz, Iran; 2 Department of Biomedical Engineering, Faculty of Engineering, University of Malaya, Kuala Lumpur, Malaysia; 3 Biomaterials Group, Nanotechnology and Advanced Materials Department, Materials and Energy Research Center, Alborz, Iran; 4 Department of Biomaterials, Science and Research Branch, Islamic Azad University, Yazd, Iran; 5 Faculty of Engineering and Natural Sciences, Sabanci University, Istanbul, Turkey; 6 Department of Chemical and Materials Engineering, University of Alberta, Edmonton, Alberta, Canada; Texas A&M University Baylor College of Dentistry, UNITED STATES

## Abstract

In the current study, a sol-gel-synthesized tricalcium magnesium silicate powder was coated on Ti-6Al-4V alloys using plasma spray method. Composition of feed powder was evaluated by X-ray diffraction technique before and after the coating process. Scanning electron microscopy and atomic force microscopy were used to study the morphology of coated substrates. The corrosion behaviors of bare and coated Ti-6Al-4V alloys were examined using potentiodynamic polarization test and electrochemical impedance spectroscopy in stimulated body fluids. Moreover, bare and coated Ti-6Al-4V alloys were characterized in vitro by culturing osteoblast and mesenchymal stem cells for several days. Results demonstrated a meaningful improvement in the corrosion resistance of Ti-6Al-4V alloys coated with tricalcium magnesium silicate compared with the bare counterparts, by showing a decrease in corrosion current density from 1.84 μA/cm^2^ to 0.31 μA/cm^2^. Furthermore, the coating substantially improved the bioactivity of Ti-6Al-4Valloys. Our study on corrosion behavior and biological response of Ti-6Al-4V alloy coated by tricalcium magnesium silicate proved that the coating has considerably enhanced safety and applicability of Ti-6Al-4V alloys, suggesting its potential use in permanent implants and artificial joints.

## Introduction

Bioceramics were used in several studies to modify the surface properties of metallic implants for the improvement of their biological and corrosion behaviors [1–4].Over the past years,Ti-6Al-4V alloy has been widely utilized in medical equipment, dental implants, bone plates, and artificial joints [3–6]. A metallic implantlikeTi-6Al-4V alloy, is corroded by exposure to body fluids which include corrosive chloride ions, resulting in metallic ion release in the body environment [7]. The presence of metallic ions like aluminum and vanadium in the body environment can cause cell intoxication and severe allergic reactions [7, 8]. In addition to this particular problem, metallic implants, including Ti-6Al-4V alloy, demonstrate weak surface bioactivity to stimulate and increase the growth rate of cells [9]. Weak bioactivity will significantly decrease the formation and growth of new bone tissue at the interface implant-bone interface [10]. Failure in proper integration of tissue and implants at the bone–implant interface causes implant loosening in long term [11]. In most of the cases, implant loosening, like in the case of artificial joints, will require a second surgery to solve the problem [12]. In order to improve the corrosion and biological properties of a metallic implant (e.g., Ti-6Al-4V alloy), bioceramic coatings like hydroxyapatite and calcium silicates were developed by a plasma spray technique [13, 14]. A well-known bioceramic like hydroxyapatite would be an excellent candidate for coating on Ti-6Al-4V substrates, by considering its superior biocompatibility and bioactivity in the body environment [14, 15]. However, the long-term chemical and physical stability of a bioceramic coating in the body environment is a major concern. The chemical and mechanical stability of coatings are fundamentally related to the dissolution rate of coatings exposed to body fluids and the coating adhesion to implant, respectively [12, 16]. The chemical and physical instability of the implant coating causes the loosening of implants, indicating the need to develop novel bioceramic coatings and methods to overcome this particular problem [17–19].

Deposition of a calcium phosphate or a calcium silicate coatings like hydroxyapatite or wollastonite on titanium alloys can result in some chemical and physical instability in vivo [19–21]. The instability of plasma-sprayed hydroxyapatite coating on titanium alloys is attributed to the presence of a tricalcium phosphate phase, which results from the phase decomposition of hydroxyapatite at high temperature during the plasma spray process [22, 23]. Hydroxyapatite can maintain its phase stability at temperatures up to 1200°C, and it will transform into tricalcium phosphate phase at temperatures higher than 1200°C; whichis highly soluble in the body environment [24].The presence of a tricalcium phosphate phase in hydroxyapatite coatings causes a higher dissolution rate of the coating in body fluids, resulting in a decrease in stability of the implant coating [22, 23]. The long-term evaluation of chemical stability of calcium silicate coatings, such as CaSiO_3_, has demonstrated undesirable results [21]. Moreover, residual thermal stresses at the interface between the bioceramic coating and metallic implant are caused by the plasma spray process, which will turn to a decrease in the mechanical stability of well-known and common coatings, such as hydroxyapatite and calcium silicates [25, 26]. The collision of high-temperature particles with the metal surface, as well as the great difference in the thermal expansion coefficient of Ti-6Al-4V alloy as the substrate (9.80×10^−6^/°C) and hydroxyapatite bioceramic (11.5×10^−6^/°C), results in high thermal stresses at the substrate–coating interface [26]. The residual stress at the coating–implant interface and the nature of fatigue loadings in the body environment will facilitate crack formation in the coating and result in the separation of the coating from the implant surface [16, 26].

Bioceramic coatings developed with desirable chemical stability in the body environment and a thermal expansion coefficient close to the substrates have been the subject of several studies [27–30]. Modification of calcium silicate bioceramics by zinc and titanium elements has demonstrated a substantial chemical stability (in terms of solubility) in the body fluids compared with common calcium silicate compounds [27]. Moreover, incorporation of magnesium in calcium silicate compounds will significantly enhance the chemical stability [28]. Razavi et. al. [31, 32] have coated a magnesium alloy substrates with merwinite to enhance its chemical stability and reported a significant increase in its bioactivity compared to the bare magnesium alloy. In addition, the thermal expansion coefficient of magnesium-incorporated calcium silicate compositions (9.87×10^−6^/°C) is close to that of Ti-6Al-4V alloy (9.80×10^−6^/°C)[29].

We have recently reported plasma spray coating of Ti-6Al-4V substrates with merwinite, emphasizing on the process parameters and microstructural features of the coating [33]. To the best of our knowledge, there are no other studies on the corrosion behavior and in vitro characterization of Ti-6Al-4V substrates coated with merwinite. In the present study, the effects of tricalcium magnesium silicate bioceramic coating on the electrochemical behavior of Ti-6Al-4V alloys were studied by potentiodynamic polarization test and electrochemical impedance spectroscopy. The biological behaviors of Ti-6Al-4V alloys coated with tricalcium magnesium silicate and their ash-coated counterparts were evaluated in osteoblast cells.

## Materials and Methods

### Electrochemical behavior

Nanostructured tricalcium magnesium silicate powders were synthesized by the sol-gel technique in our prior study [34]. After granulation, the obtained powder was sieved through 80 mesh. The Ti-6Al-4V substrate with dimensions of 1 cm × 1.5 cm × 0.2 cm was ultrasonically grit blasted. After washing with ethanol and drying at 60°C, the atmosphere plasma spray system (Sulzer Metco, Switzerland) was used to coat the materials according to the parameters in Table 1.

**Table 1:** Detailed parameters for preparing plasma-sprayed coatings [33].

Parameters of plasma spray procedure	Content
**Argon plasma gas flow rate (slpm)**	**85**
**Hydrogen plasma gas flow rate (slpm)**	**10**
**Spray distance (mm)**	**100**
**Argon powder carrier gas (slpm)**	**10**
**Current (A)**	**400**
**Voltage (V)**	**55**
**Powder feed rate (gr min** ^**−1**^ **)**	**9**

The potentiodynamic polarization test and electrochemical impedance spectroscopy (IVIUM, 5612Ajeindoven, Netherlands) were used to evaluate the effect of tricalcium magnesium silicate coating on the electrochemical behavior of Ti-6Al-4V alloys. A saturated calomel electrode (Ag/AgCl) and platinum electrode were utilized as the reference and auxiliary electrodes, respectively. Experiments were performed in the simulated body fluid (SBF) at 37°C according to Table 2 [35]. The scan rate used for the polarization test was 1 mV/s. The electrochemical parameters like the corrosion current density (*i*_corr_), polarization resistance (*R*_p_), and corrosion potential (*E*_corr_), were extracted from the extrapolation of polarization curves. The electrochemical impedance was measured in the open circuit potential with an applied frequency ranging from100 kHz to 10 mHz by applying a sinewave with an amplitude of 10 mV.

**Table 2:** Composition of SBF fluid [35].

Reagent	Amount	Purity%	Formula weight
**NaCl**	**8.036 gr**	**99.5**	**58.44**
**NaHCO**_**3**_	**0.352 gr**	**99.7**	**84.01**
**KCl**	**0.225 gr**	**99.0**	**74.56**
**K**_**2**_ **HPO**_**4**_ **.3H**_**2**_ **O**	**0.230 gr**	**99.0**	**228.23**
**MgCl**_**2**_ **.6H**_**2**_ **O**	**0.311 gr**	**99.0**	**203.30**
**1 M HCl**	**40 ml**	**-**	**-**
**CaCl**_**2**_	**0.293 gr**	**98**	**110.98**
**Na**_**2**_ **SO**_**4**_	**0.072 gr**	**99.0**	**142.04**
**Tris**	**6.063 gr**	**99.8**	**121.14**
**1 M HCl**	**0.2 ml – 0**	**-**	**-**

### Biological behavior

Cellular tests were employed in order to study the impact of the tricalcium magnesium silicate coating on the biological behavior of Ti-6Al-4V alloy. Cell proliferation was studied using the MTT assay (Sigma Inc., St. Louis, MO, USA). Optical and scanning electron microscopy (SEM) were used to assess the morphology of cells at the sample-medium interface and cell morphologies on the sample surface, respectively. Samples were sterilized at 200°C for 2 h in the autoclave. Three groups of cells were cultured: one in the presence of the Ti-6Al-4V alloy substrate, another in the presence of the coated Ti-6Al-4V alloy, and the third without a metallic sample as the control. The cells were cultured in a 24-well plate.

Cells were seeded at an initial density of 10^5^ cells/well, suspended in 1 mL of Dulbecco’s modified Eagle’s medium (DMEM) supplemented with 10% fetal bovine serum (FBS) and 1% penicillin streptomycin in the culture flasks. Cultures were incubated at 37°C in an atmosphere of 5% CO_2_. Subsequently, the cultures were altered and frequently controlled in the following days.

Osteoblast G292 cell line and bone marrow mesenchymal stem cells (MSCs) (MSCs were isolated from Male Wistar rats) were purchased from Pasteur Institute of Iran (National Cell Bank of Iran, Pasteur Institute of Iran, Tehran, Iran).The direct cell test was employed to investigate the effect of theTi-6Al-4V alloy coated with tricalcium magnesium silicate on osteoblast cells. An optical microscope (1X832model; Deck Inverted Microscope) was used to monitor the culture, cell density, and sample–culture interface after 72 h. The MTT assay was used to evaluate the proliferation of osteoblast G292 cells in the first, second, and third days of culture. About 5 mg/mL MTT solution in PBS was prepared.

Subsequently, 100 μL of MTT was added to 900 μL of DMEM/F12 for dilution. To each sample in a 24-well plate, 1 mL of the diluted MTT solution was added. After 2 h of incubation, 1 mL of the stabilized solution containing 10% Triton X-100, 0.1 M HCl, and isopropanol was added to dissolve the formazan crystals. The medium was shaken for 10 min before a 100 mg aliquot was examined with a USA STATEAX apparatus to measure the absorption rate and a microplate reader at a wavelength of 570 nm.

To study the cell morphology of the samples, approximately 10,000mesenchymal stem cells of the bone marrow from each sample, which had been passaged for four times and counted with a hemocytometer, were diluted in 1 mL of DMEM supplemented with 10% FBS and 1% penicillin–streptomycin. The cells were incubated in a24-well plate for 72 h and then cultured in an incubator at 37°C under an atmosphere of 5% CO_2_. The samples were washed twice with PBS solution. The culture medium of the samples was changed every 24 h. After 48 h, the cells were stabilized with 2.5% glutaraldehyde solution for 24 h at 4°C. Subsequently, the surface morphology of all samples was studied by SEM after dehydration in 10%, 30%, 50%, 70%, 80%, 90%, 95%, and 100% ethanol.

Measurements were done for three sets of samples in electrochemical and biological tests and the average was reported. Microsoft Excel software (Microsoft, USA) was used for data processing.

### Characterization

The microstructure of the powder, which was synthesized by the sol-gel technique, was studied by transmission electron microscopy (TEM, CM 20; Philips, Netherlands). Phase analysis of the synthesized powder and tricalcium magnesium silicate coating has been studied by X-ray diffraction analysis (XRD, Advance D8; Bruker, Germany). In addition, the morphology and topography of the tricalcium magnesium silicate coating were evaluated by SEM (XL30; Philips, Netherlands) and atomic force microscopy (AFM; Philips, Netherlands), respectively. Moreover, energy dispersive X-ray analysis (EDX) was employed for the elemental analysis of the tricalcium magnesium silicate coating.

## Results

**Fig 1:**
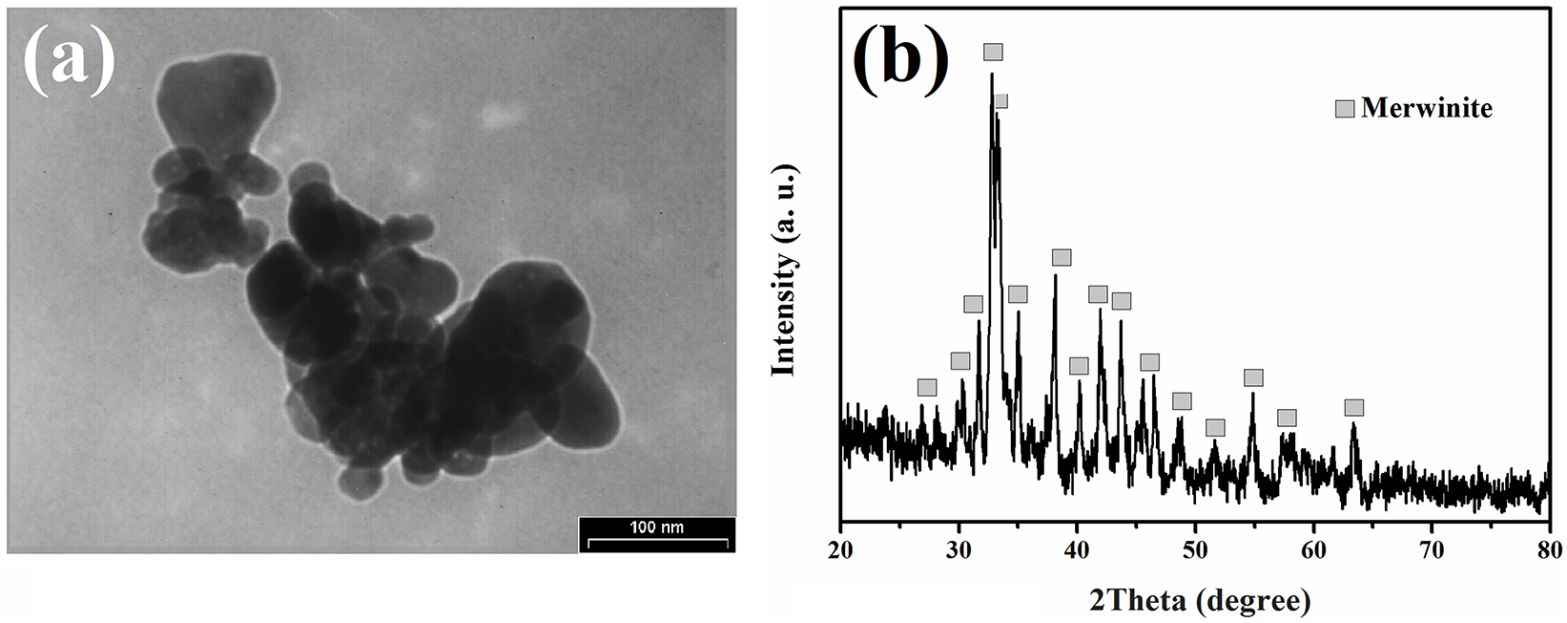
TEM image (a) and X-ray diffraction pattern (b) of the tricalcium magnesium silicate powder.

Fig 1(A) and 1(B) present the TEM image and XRD pattern of the tricalcium magnesium silicate powder, which was synthesized by the sol-gel technique. The TEM image of the synthesized tricalcium magnesium silicate powder shows the crystallite size in the range of less than 100 nm. As shown in the XRD pattern, the tricalcium magnesium silicate phase was in the nanoscale but possessed a high degree of crystallinity.

**Fig 2:**
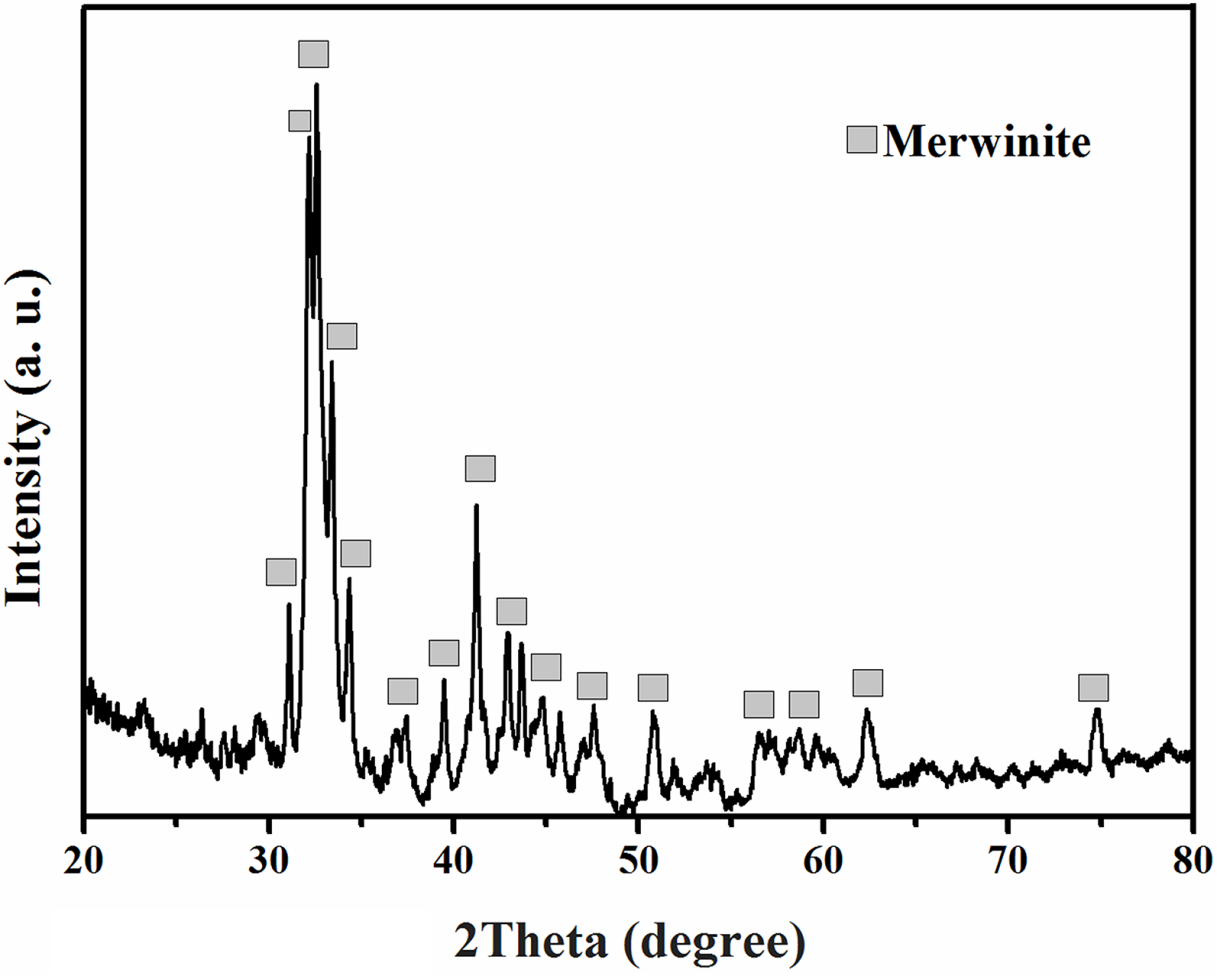
X-ray diffraction pattern of the tricalcium magnesium silicate coating.

**Fig 3:**
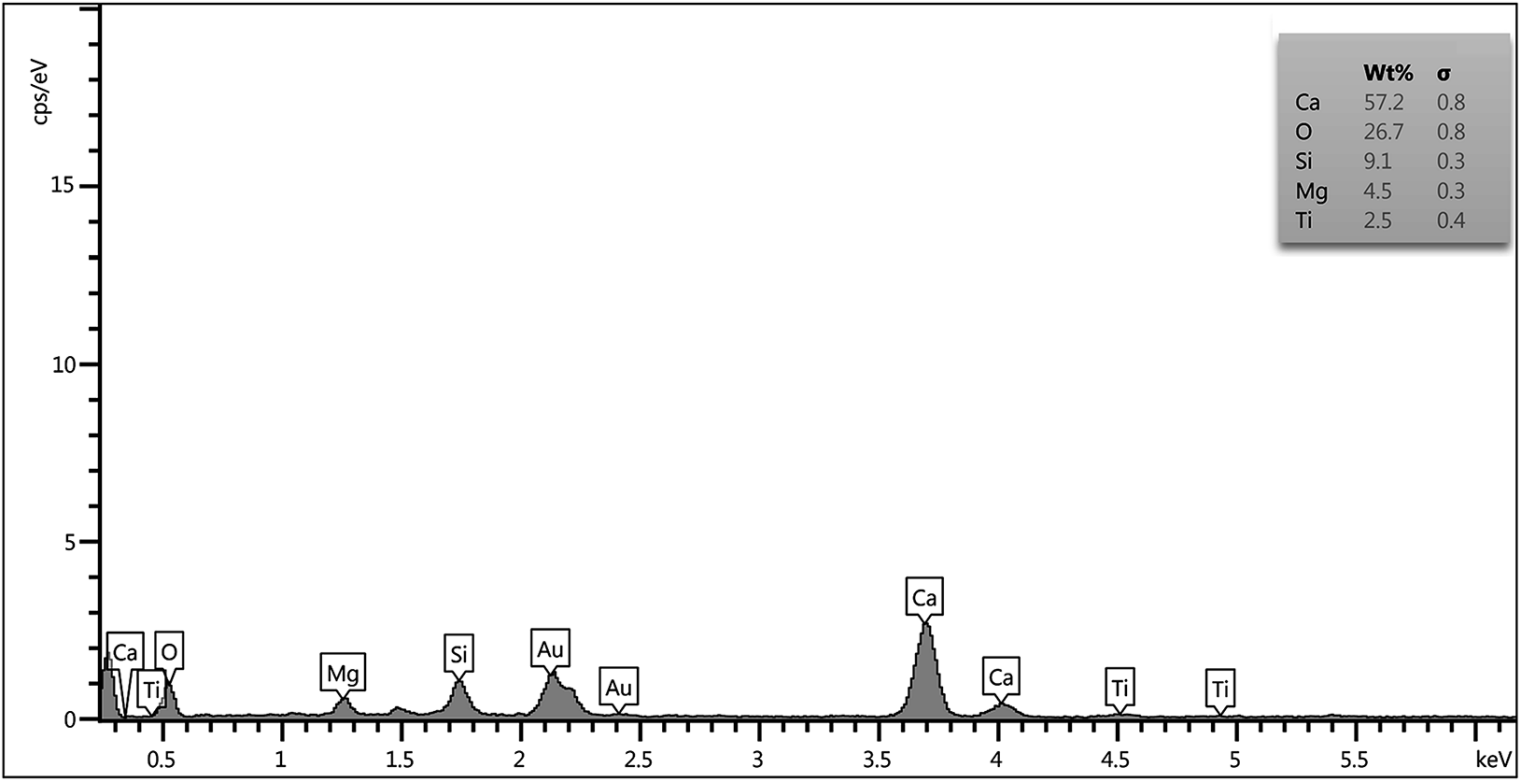
EDX analysis of the tricalcium magnesium silicate coating.

Fig 2 illustrates the XRD pattern of the tricalcium magnesium silicate powder, which was coated on the Ti-6Al-4V alloy by the plasma spray technique. The peaks related to the tricalcium magnesium silicate phase were identified in the XRD pattern. Moreover, the presence of a tricalcium magnesium silicate phase was semi-quantitatively identified with respect to EDX analysis, as shown in Fig 3.

**Fig 4:**
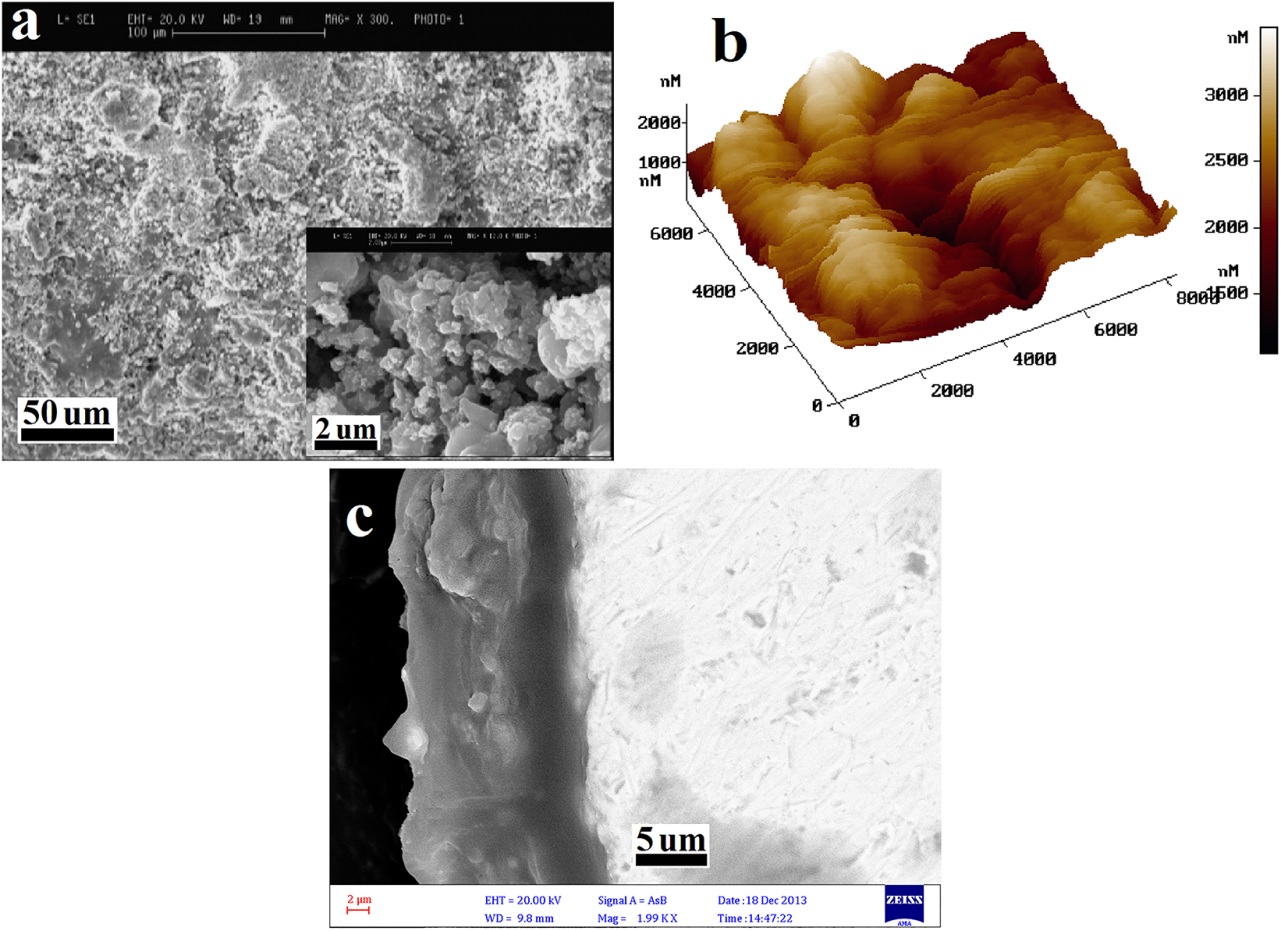
SEM (a), AFM (b), and cross-section SEM (c) images of the tricalcium magnesium silicate coating.

The SEM and AFM images of the tricalcium magnesium silicate coating are also depicted in Fig 4(A) and 4(B), respectively. Moreover, the cross-section SEM image of the Ti-6Al-4V alloy coated by tricalcium magnesium silicate is depicted in Fig 4(C). A homogenous tricalcium magnesium silicate coating was present on the Ti-6Al-4V alloy, with an average thickness of 16 μm.

**Fig 5:**
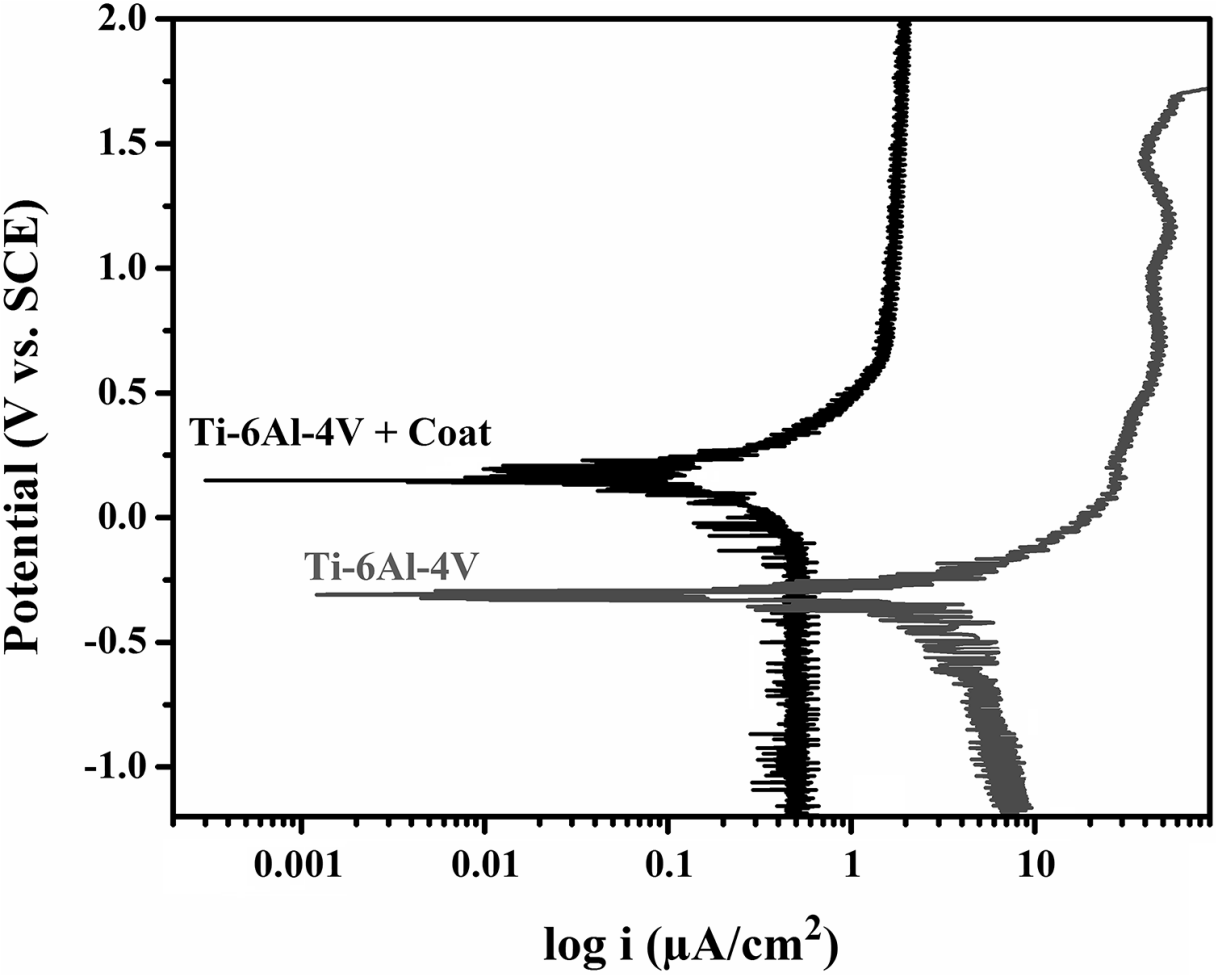
Polarization test curves of the bare Ti-6Al-4V alloy and the Ti-6Al-4V alloy coated with tricalcium magnesium silicate.

Fig 5 displays the polarization test curves of the uncoatedTi-6Al-4V and the Ti-6Al-4V alloy that was coated by tricalcium magnesium silicate. Table 3 reveals the electrochemical parameters, such as *R*_p_, *i*_corr_, and *E*_corr_, which were found by the extrapolation of polarization curves. The results of the polarization test showed that the corrosion rate of the Ti-6Al-4V alloy decreased by more than five times after coating with tricalcium magnesium silicate.

**Table 3:** Electrochemical data from the polarization curves.

Sample	E_corr_ (mV vs. SCE)	I_corr_ (μA.cm^-2^)	R_p_ (MΩ.cm^2^)
**Ti-6Al-4V**	**-309**	**1.84**	**0.47**
**Ti-6Al-4V + Coat**	**+216**	**0.31**	**0.73**

**Fig 6:**
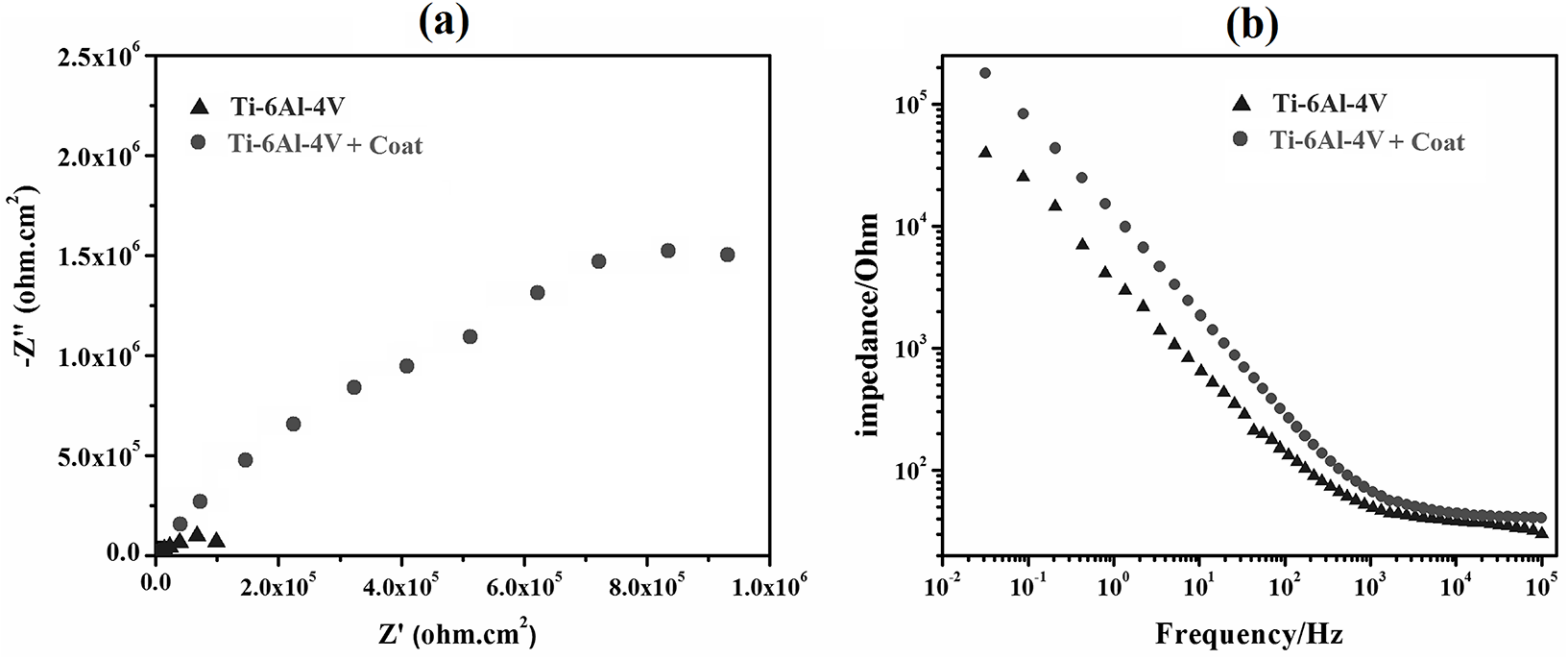
Nyquist (a) and Bode (b) plots of the bare Ti-6Al-4V alloy and theTi-6Al-4V alloy coated with tricalcium magnesium silicate.

**Fig 7:**
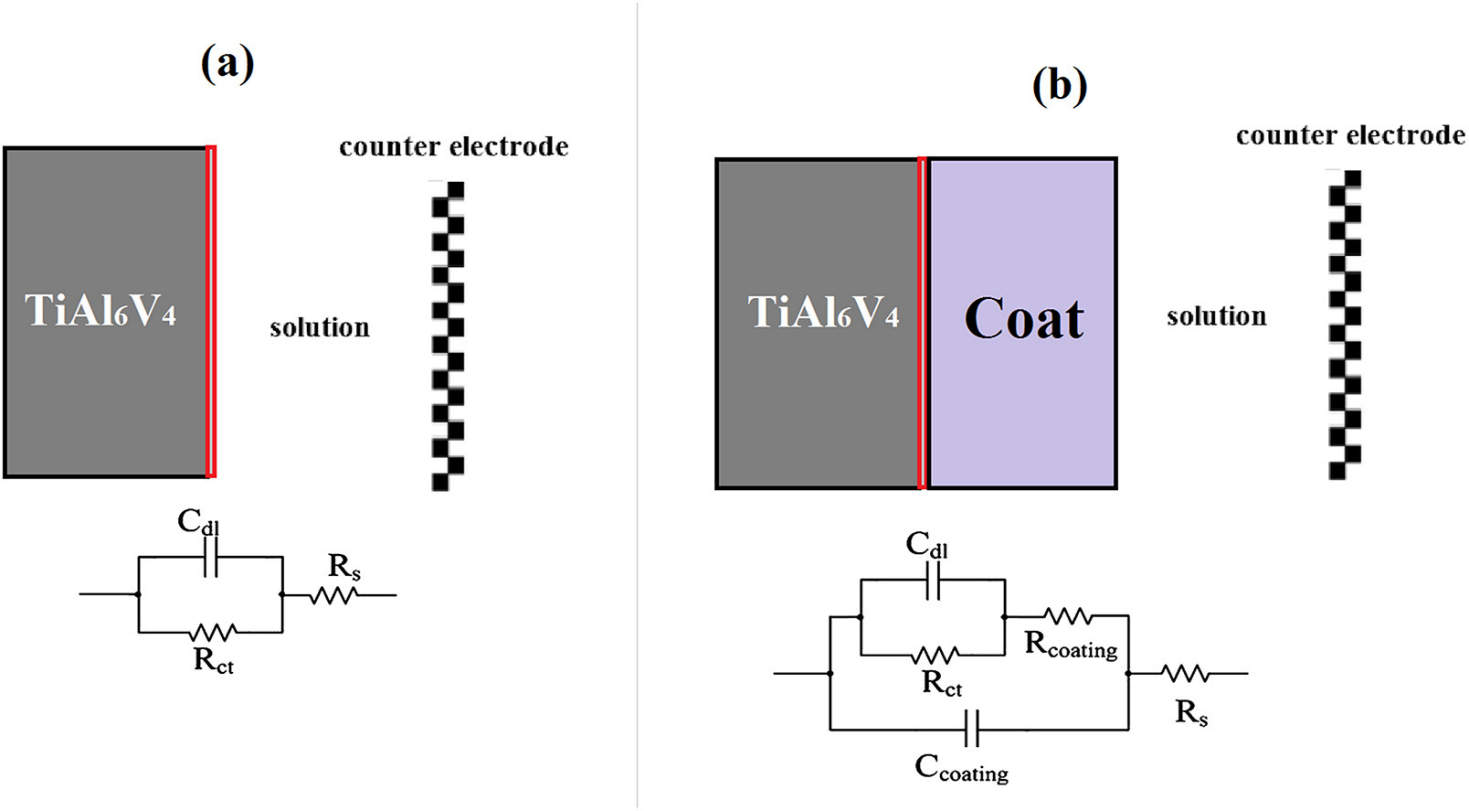
Equivalent circuit of EIS curves of the bare (a) and coated (b) Ti-6Al-4V alloys.

Fig 6(A) and 6(B) demonstrate the Nyquist and Bode plots of the Ti-6Al-4V alloy coated with tricalcium magnesium silicate and the as-coated alloy as found in the EIS test, respectively. The equivalent circuit of the EIS curves is shown in Fig 7, in which (a) and (b) are related to the bare and coated Ti-6Al-4V alloys, respectively. An electrical double layer was created on the sample surface, which determined the polarization resistance of the sample when it was immersed in SBF. By increasing the resistance of the electrical double layer, the sample surface showed more resistance against the penetration of corrosive ions, such as Cl^-^, onto the metal substrate [16].

The equivalent circuit is shown in Fig 7(B), which includes the electrolyte resistance (*R*_s_), coating resistance against the charge transfer (*R*_coat_), coating capacitance (*C*_coat_), charge transfer resistance of double layer at the Ti-6Al-4V-coating interface (*R*_ct_), and double layer capacitance (*C*_dl_). Table 4 shows the values of the equivalent circuit related to the datawhich were extracted from the EIS test by the ZSimpWin software. According to the values presented in Table 4, the presence of a tricalcium magnesium silicate coating significantly increased the corrosion resistance of the Ti-6Al-4V alloy. Thus, the tricalcium magnesium silicate bioceramic coating served as a barrier against the penetration of SBF ions onto the metallic substrate. This layer could be considered a resistance factor against the anodic and cathodic reactions on the metal surface

**Table 4:** Electrochemical parameters extracted from EIS data.

Sample	R_s_ (Ω)	R_ct_ (MΩ.cm^2^)	C_dl_ (μF/cm^2^)	R_coating_ (KΩ.cm^2^)	C_coating_ (μF/cm^2^)
**Ti-6Al-4V**	**54**	**0.048**	**10.02**	**-**	**-**
**Ti-6Al-4V + Coat**	**56**	**0.724**	**8.17**	**135**	**12.19**

**Fig 8:**
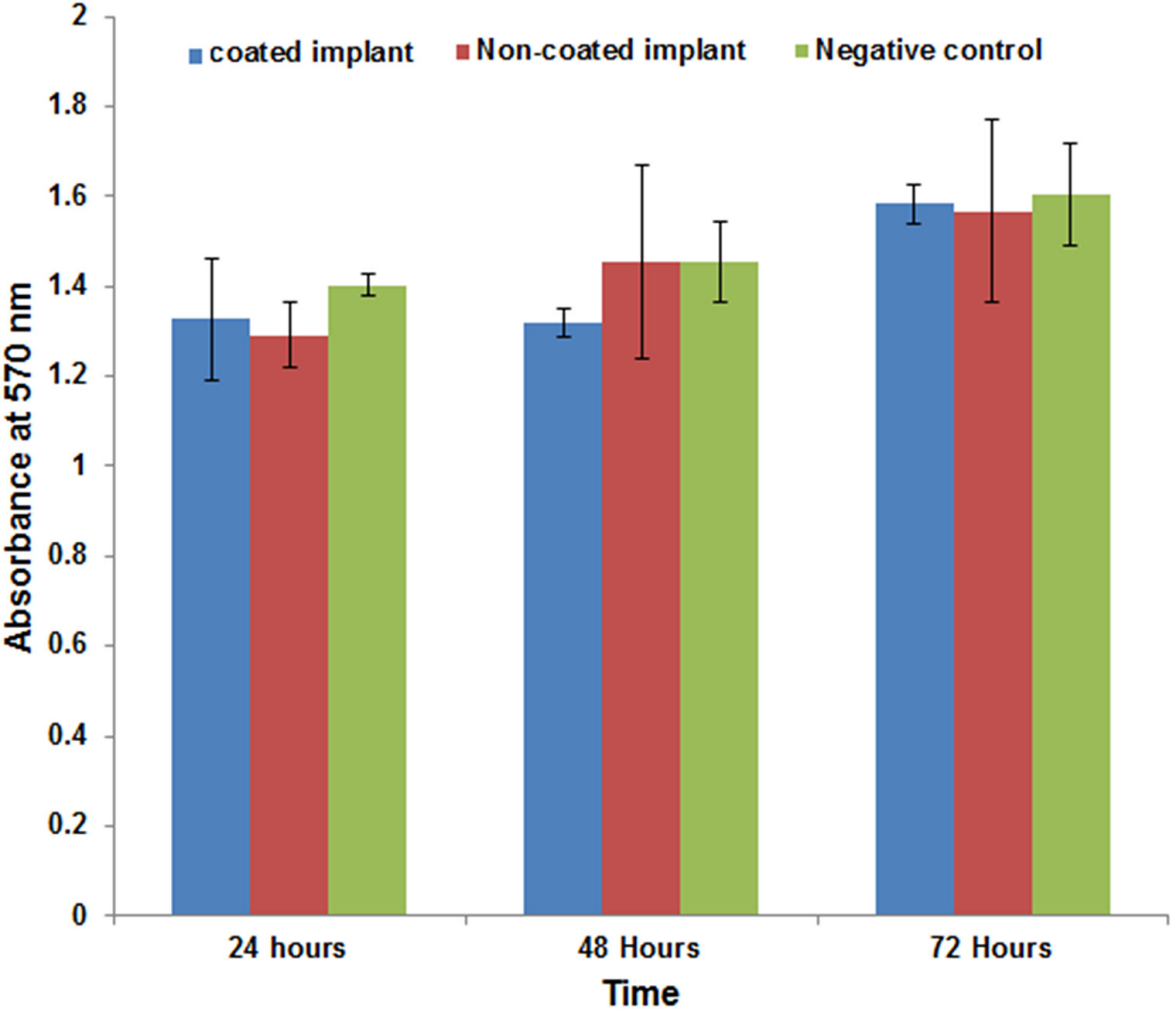
MTT assay results.

The results of MTT assays on osteoblast cells are presented in Fig 8. The Ti-6Al-4V alloy coated by tricalcium magnesium silicate showed no significant toxic effects, and cell proliferation did not decrease after 72 hours of incubation.

**Fig 9:**
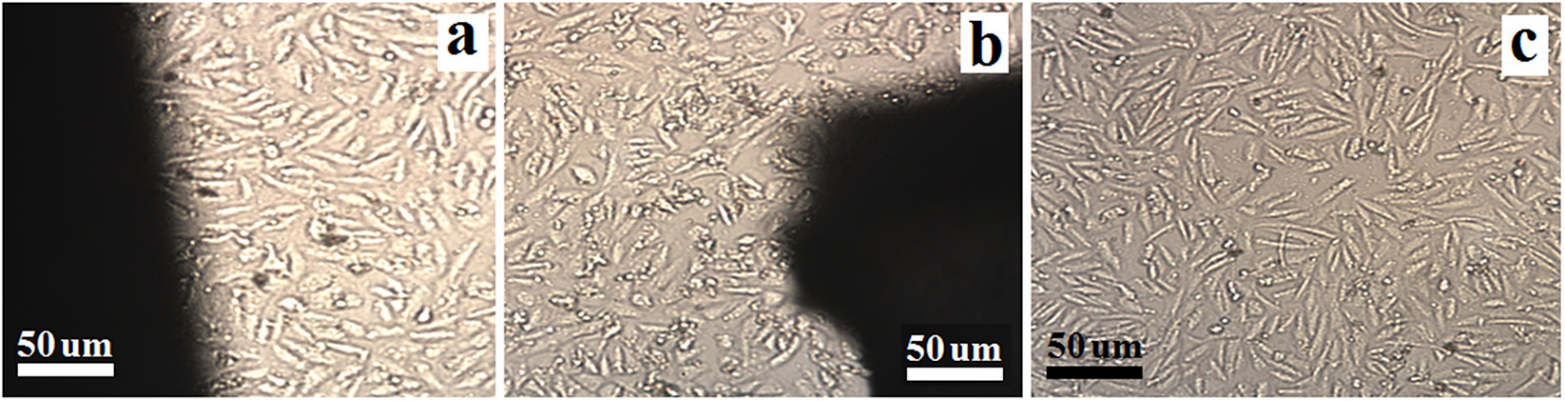
Cell morphology after cell growth of cultures with the bare Ti-6Al-4V alloy (a), the coated Ti-6Al-4V alloy (b), and the culture media (c) after 72 h.

Fig 9 presents a micrograph of the direct contact test of cells after 72 h. No differences were observed in the morphology and cell density adjacent to the Ti-6Al-4V alloy coated with tricalcium magnesium silicate compared with the Ti-6Al-4V alloy. Fig 9 demonstrates that the cell morphology was fusiform after the cell growth stage. The culture with the Ti-6Al-4V alloy coated with tricalcium magnesium silicate had no cytotoxic effects.

**Fig 10:**
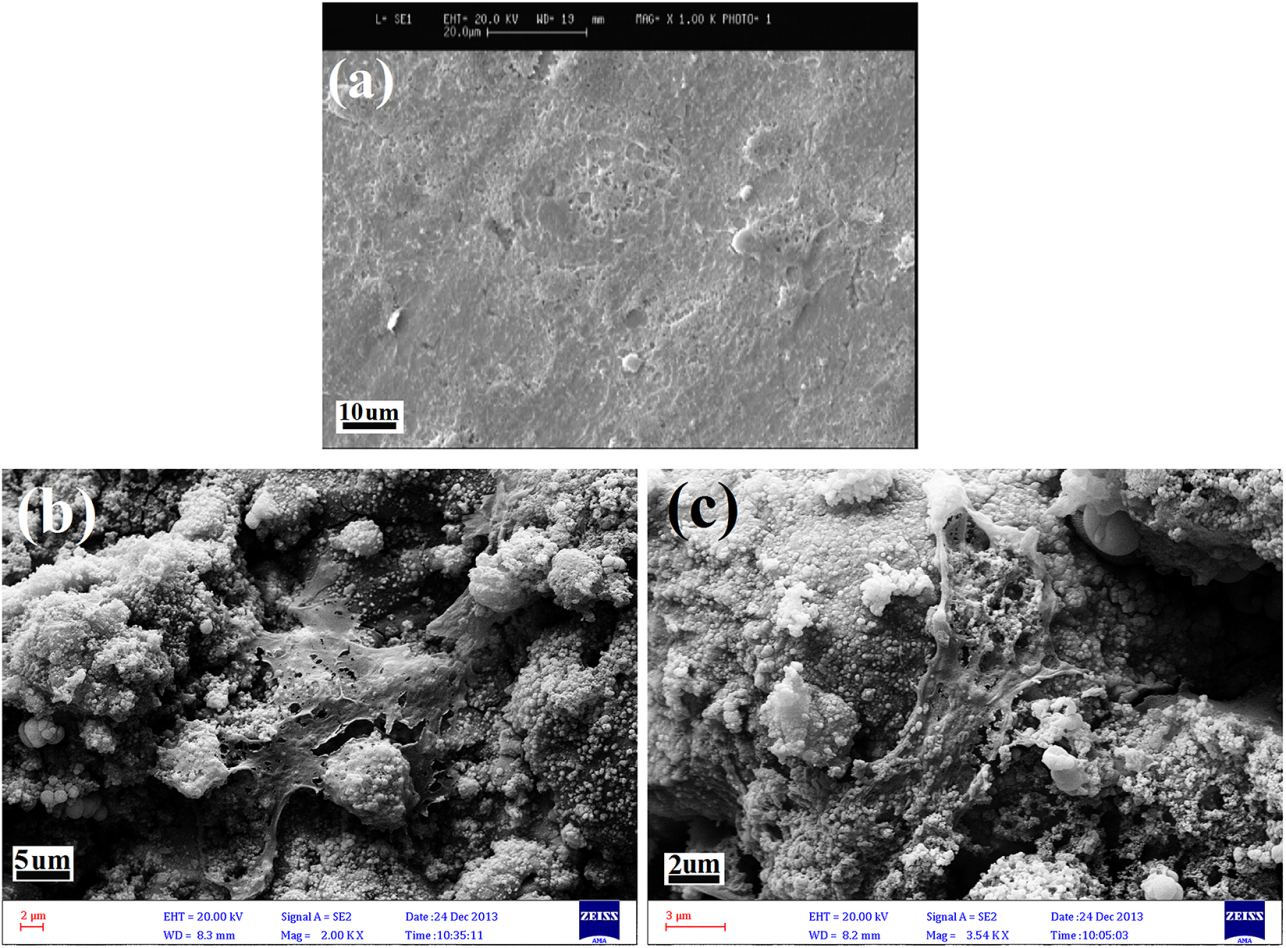
Morphology of the cells on the surface of the bare Ti-6Al-4V alloy (a) and the Ti-6Al-4V alloy coated with tricalcium magnesium silicate (b, c) after 5 d of culture.

Fig 10 illustrates the morphology of the cells on the surface of the bare Ti-6Al-4V alloy and the Ti-6Al-4V alloy coated with tricalcium magnesium silicate after 5 d of culture. As shown in Fig 10, the cells were homogeneously and more widely distributed on the surface of the tricalcium magnesium silicate coating compared with the bare Ti-6Al-4V alloy.

### Discussion

In the current study, thetricalcium magnesium silicate coating showed great phase stability, and only a negligible portion of the coating was transformed into the amorphous phase. Unlike hydroxyapatite and other common bioceramics, the thermal expansion coefficient of tricalcium magnesium silicate is very close to that of the Ti-6Al-4Valloy [29].This relative similarity between the thermal expansion coefficient of the bioceramic and metallic substrate resulted in less formation of longitudinal cracks in the coating, thereby increasing the stability of the coating/substrate bonds [29]. By contrast, previous studies reported the formation of some longitudinal cracks in bioceramic coatings, such as hydroxyapatite and akermanite, on Ti-6Al-4V alloys, which were coated via the plasma spray technique [36].The formation of cracks in bioceramic coatings like akermanite during the coating process is attributed to a significant difference between the thermal expansion coefficient of the coating and Ti-6Al-4V alloy; thus, the stress at the coating–substrate interface considerably increases, which eventually causes the production of longitudinal cracks[26, 29]. The cross-sectional SEM image of the tricalcium magnesium silicate coating on the Ti-6Al-4V alloy in Fig 4(C) demonstrates a uniform coating without any cracks at the interface.

Yi et al. [37] have previously coated akermanite on Ti-6Al-4V alloy by using plasma spray technique. Akermanite, which is a member of calcium magnesium silicate family, is considered as a bioactive ceramic with excellent biological behavior. However, the difference between the thermal expansion coefficient of Ti-6Al-4V alloy and akermanite will result in the presence of residual stresses at the ceramic coating/substrate interface, inducing the formation of microcracks and leading to instability of coating on the metal surface in the long term. Yi et. al 37] confirmed the presence of microcracks in cross sections of substrates coated with akermanite by plasma spray technique in their reported SEM images.

Razavi et. al [31] coated calcium magnesium silicate (merwinite) on the magnesium alloy for the first time, by employing the electrophoretic deposition (EPD) technique. They reported a substantial improvement in corrosion resistance of magnesium alloy coated by merwinite compared to akermanite [31]. In addition, Razavi and colleagues [32] investigated the biological behavior of merwinite coating on magnesium alloy, reported that merwinite coating possesses a significant bioactivity property. Undoubtedly, improved bioactivity of the implant’s surface will consequentially accelerates the new bone formation at the implant/bone interface, and will significantly stabilize the bond between the implant and bone (like in the case of hip joint implants) in the body environment [12, 24].

The presence of a tricalcium magnesium silicate coating on the Ti-6Al-4V alloy increased the cellular interaction with the metallic substrate. The tricalcium magnesium silicate coating significantly improved the biological behavior of the Ti-6Al-4V alloy, and enhanced the adhesion and distribution of osteoblast cells on the surface of the alloy.

Ou et al. [29] reported the mechanical properties of merwinite and compared their findings with those of natural bone. Similarity between Young elastic modulus of merwinite and natural bone indicates the potentially good mechanical biocompatibility of merwinite bioceramics. Furthermore, they claimed that presence of silicon is the main reason for increased proliferation of cells on merwinite’s surface [32, 29].Also, Razavi et. al [31, 32]showed that the formation of surface Si–OH groups on merwinite plays a key role in possessing its superior bioactivity.

Additionally, the selection of a nano-sized tricalcium magnesium silicate powder have improved the chemical and mechanical behavior of the coating, by partial melting of nanoparticles during the plasma spray process, which could be considered as the cause for increasing the coating crystallinity[38, 39].

### Conclusion

In the current study, tricalcium magnesium silicate is suggested as a novel coating material to improve the corrosion and biological behavior of permanent metallic implants. Aside from its high stability in the body environment, tricalcium magnesium silicate has almost the same thermal expansion properties as the Ti-6Al-4V alloy, which eventually forms stable bonds at the bioceramic–alloy interface. In this study, nano-sized tricalcium magnesium silicate powder was used to coat the Ti-6Al-4V alloys via the plasma spray method. Deposited coating layer was uniform, with a thickness of 40 μm. The presence of tricalcium magnesium silicate coating on Ti-6Al-4V alloy substantially improved the corrosion resistance, whereas the corrosion current density of the Ti-6Al-4V alloy sample declined from 1.84 μA/cm^2^ to 0.31 μA/cm^2^. As a result, significant improvement in biological behavior was observed, specifically an enhancement in the distribution and proliferation of cells cultured on the Ti-6Al-4V alloy after coating with tricalcium magnesium silicate bioceramic.
